# Pharmacogenetic Review: Germline Genetic Variants Possessing Increased Cancer Risk With Clinically Actionable Therapeutic Relationships

**DOI:** 10.3389/fgene.2022.857120

**Published:** 2022-05-24

**Authors:** Austin A. Saugstad, Natasha Petry, Catherine Hajek

**Affiliations:** ^1^ Kansas City University, College of Osteopathic Medicine, Kansas City, MO, United States; ^2^ Sanford Health Imagenetics, Sioux Falls, SD, United States; ^3^ Department of Pharmacy Practice, College of Health Professions, North Dakota State University, Fargo, ND, United States; ^4^ University of South Dakota, Sanford School of Medicine, Department of Internal Medicine, Sioux Falls, SD, United States

**Keywords:** pharmacogenomics, pharmacogenetics, germline, gene-drug relationships, Cancer predisposition gene

## Abstract

As our understanding of genomics and genetic testing continues to advance, the personalization of medical decision making is progressing simultaneously. By carefully crafting medical care to fit the specific needs of the individual, patients can experience better long-term outcomes, reduced toxicities, and improved healthcare experiences. Genetic tests are frequently ordered to help diagnose a clinical presentation and even to guide surveillance. Through persistent investigation, studies have begun to delineate further therapeutic implications based upon unique relationships with genetic variants. In this review, a pre-emptive approach is taken to understand the existing evidence of relationships between specific genetic variants and available therapies. The review revealed an array of diverse relationships, ranging from well-documented clinical approaches to investigative findings with potential for future application. Therapeutic agents identified in the study ranged from highly specific targeted therapies to agents possessing similar risk factors as a genetic variant. Working in conjunction with national standardized treatment approaches, it is critical that physicians appropriately consider these relationships when developing personalized treatment plans for their patients.

## Introduction

Advances in both availability and affordability of genetic screening, in conjunction with continued study of therapeutic interactions, has made the utilization of genetic data in clinical practice of the utmost importance. As the usage of genetic information to guide medical decision making continues to increase in frequency, patients are beginning to receive more precise clinical care. Through the utilization of patient-specific genetic information in the lens of physician-guided clinical context, patients are experiencing improved long-term outcomes, reduced toxicities, and fewer adverse effects (van der Wouden et al., 2019; Bernard et al., 2021).

The purpose of this review is to build upon and contribute to the future utilization of genetics in the clinical setting. This study takes a pre-emptive approach, reviewing evidence of key relationships between known cancer predisposing germline genetic variants and various therapies. Our review examines 23 individual genes each with an association to one of 15 different familial cancer syndromes, in concordance with the 2017 ACMG59 recommendation list (Kalia et al., 2017). As summarized in Table 1, our review uncovers a wide array of unique therapeutic interactions between individual patient genetics and pharmacologic interventions. Evidence identified in this review is specific to germline variants and does not include findings involving somatic or therapy-induced genetic variants. In total, our review presents 54 relationships, providing clinicians and researchers with a foundational platform for the future and contributing a useful summary of existing gene-drug relationships for cancer predisposing germline variants.

**TABLE 1:** Gene-drug relationships.

Gene	Drug	Relationship	Articles	Study method	Germline Vs Tumor Derived	Score
APC	EGFR-inhibitors	Sensitivity	Yang et al. (2019)	In vitro cell lines	Tumor derived	2/4
	TNKS-inhibitors	Sensitivity	Schatoff et al. (2019)	In vivo models and human cell lines	Tumor derived	2/4
			Tanakaa et al. (2017)	Genomic scoring of clinical trial patient data, in vitro cell lines	Tumor derived	
	Aspirin and NSAIDs	Shared risk outcome	FDA, (2018)	FDA label; Two randomized clinical trials	Germline	4/4
		Sensitivity	Burn et al. (2011)	Randomized controlled trial	Germline	4/4
			Ishikawa et al. (2021)	Randomized controlled trial	Germline	
			Samadder et al. (2018)	Secondary analysis of a randomized clinical trial	Germline	
BRCA1/2	Oral Contraceptives	Clinical context consideration	Whittemore et al. (2004)	Retrospective study	Germline	4/4
			Narod et al. (1998, 2001a)	Case-control questionnaire	Germline	
			McGuire et al. (2004)	Case-control study	Germline	
			Iodice et al. (2010)	Meta-analysis	Germline	
			Huber et al. (2020)	Review	Germline	
	Clomiphene Citrate	Shared risk outcome	FDA, (2012)	FDA label	Not specific	1/4
			Reigstad et al. (2017)	Population study	Not specific	
	Estrogen-based replacement therapy	Clinical context consideration	Kotsopoulos et al. (2016)	Observational study	Germline	3/4
			Eisen et al. (2008)	Case-control study	Germline	
			Domchek et al. (2011)	Case-control study	Germline	
			Gordhandas et al. (2019)	Review	Germline	
	Platinum compounds and Triple negative breast cancer	Sensitivity	Tutt et al. (2018)	Phase 3 Clinical Trial	Germline	4/4
			Caramelo et al. (2019)	Meta-analysis	Germline	
			Silver et al. (2010)	Phase 2 Clinical Trial (BRCA1-specific)	Germline	
			Byrski et al. (2014)	Phase 2 Clinical Trial (BRCA1-specific)	Germline	
			Hahnen et al. (2017)	Secondary analysis of clinical trial	Germline	
	PARP-inhibitors	Sensitivity	Farmer et al. (2005)	In vitro cell lines	Germline	4/4
			Madariaga et al. (2020)	Review	Germline	
			Robson et al. (2017)	Phase 3 Clinical Trial	Germline	
			Litton et al. (2018)	Phase 3 Clinical Trial	Germline	
			Golan et al. (2019)	Phase 3 Clinical Trial	Germline	
			Reiss et al. (2021)	Phase 2 Clinical Trial	Germline	
STK11	PD-1 axis inhibitors	Resistance	Skoulidis et al. (2015)	Genomic analysis of tumor biopsies	Tumor derived	2/4
			Skoulidis et al. (2018)	Retrospective cohort study	Tumor derived	
			Schoenfeld et al. (2020)	Genomic analysis of tumor biopsies	Tumor derived	
			Laderian et al.	Case series	Tumor derived	
	ERK-inhibitors	Sensitivity	Caiola et al. (2020)	In vitro and in vivo	Tumor derived	2/4
TSC1/2	Estrogen based medications, including oral contraceptives	Shared risk outcome	Yano, (2002)	Case Report	Germline	1/4
			Oberstein et al. (2003)	Case series - questionnaire	Germline	
			Yu et al. (2009)	In vitro and vivo	Germline	
			Yu et al. (2004)	In vitro cell lines	Germline	
TP53	Genotoxic chemotherapy agents	Resistance	Frebourg et al. (2020)	Review	Germline	2/4
			Kasper et al. (2018)	In vivo models	Germline	
	Chemotherapy and Chronic Lymphocytic Leukemia	Resistance	Döhner et al. (1995)	Phase 3 Clinical Trial	Not specific	4/4
			Hallek et al. (2010)	Phase 3 Clinical Trial	Not specific	
			Hallek (2019)	Review	Not specific	
	Chemotherapy and Mantle Cell Lymphoma	Resistance	Eskelund et al. (2017)	Genomic analysis of tumor biopsies	Tumor derived	2/4
	Carboplatin and Breast Cancer	Sensitivity	Sheng et al. (2020)	Cohort Study	Germline	3/4
	5-FU and Colon Cancer	Resistance	Aghabozorgi et al. (2020)	Review	Not specific	3/4
PTEN	Tamoxifen	Shared risk outcome	Hobert and Eng, (2009)	Review	Yes	1/4
	Trastuzumab and Lapatinib	Resistance	Berns et al. (2007)	In vitro cell lines	Tumor derived	2/4
			Nagata et al. (2004)	In vitro and vivo	Tumor derived	
			Wang et al. (2011)	Expanded access clinical trial	Tumor derived	
			Eichhorn et al. (2008)	In vitro and in vivo	Tumor derived	
	CDK4/6 inhibitors	Resistance	Costa et al. (2020)	In vitro and in vivo	Tumor derived	2/4
	PI3K-alpha inhibitors	Resistance	Juric et al. (2015)	Case report	Tumor derived	2/4
			Costa et al. (2020)	In vitro and in vivo	Tumor derived	
VHL	HIF-2a inhibitors	Sensitivity	Chen et al. (2016)	Patient-derived tumorgraft	Tumor derived	4/4
			Courtney et al. (2020)	Phase 1 Clinical Trial	Germline	
			Choueiri et al.	Phase 1 Clinical Trial	Germline	
			Jonasch et al. (2021)	Phase 2 Clinical Trial	Germline	
MUTYH	Alkylating agents	Resistance	Fry et al. (2008)	In vitro cell lines	Tumor derived	2/4
RET	GLP-1 agonists	Shared risk outcome	Bjerre Knudsen et al. (2010)	In vitro and in vivo	Tumor derived	2/4
			Madsen et al. (2012)	In vitro and in vivo	Tumor derived	
			FDA, (2010)	FDA label	Not specific	
	Tyrosine Kinase Inhibitors	Sensitivity	O'Kane et al. (2019)	Phase 2 clinical trial	Germline	4/4
			Wells et al. (2012)	Phase 3 clinical trial	Germline	
			Elisei et al. (2013)	Phase 3 clinical trial	Germline	
			Wirth et al. (2020)	Phase 1/2 clinical trial	Germline	
			Subbian et al. (2018)	In vitro and in vivo, two case reports	Tumor derived	
			Subbian et al. (2021)	Phase 1/2 clinical trial	Germline	
SMAD4	5-FU	Resistance	Wasserman et al. (2019)	Cohort study	Tumor derived	2/4
			Alhopuro et al. (2005)	In vitro cell lines	Tumor derived	
			Papageorgis et al. (2011)	In vitro cell lines	Tumor derived	
			Wong et al. (2020)	In vitro and in vivo	Tumor derived	
	Cetuximab	Resistance	Lin et al. (2019)	In vitro cell lines	Tumor derived	2/4
			Ozawa et al. (2017)	In vitro and in vivo	Tumor derived	
			Mei et al.	In vivo models	Tumor derived	
	Irinotecan	Resistance	Wong et al. (2020)	In vitro and in vivo	Tumor derived	2/4
	Fluoroquinolones	Shared risk outcome	FDA warning	Cites 4 published observational studies	Not specific	1/4
			CIPRO	FDA label	Not specific	
SDHB	Temozolomide	Sensitivity	Hadoux et al. (2014)	Retrospective population study	Germline	2/4
			Pang et al. (2018)	In vitro and in vivo, patient samples	Tumor derived	
	Tyrosine Kinase Inhibitors	Resistance (GISTs)	Boikos et al. (2016)	Observational study	Germline	2/4
			Paik et al. (2014)	Case Report	Germline	
		Sensitivity (Metastatic PGG and PCC)	O'Kane et al. (2019)	Phase 2 clinical trial	Germline	3/4
SDHA	Tyrosine Kinase Inhibitors	Resistance (GISTs)	Boikos et al. (2016)	Observational study	Germline	2/4
		Sensitivity (Metastatic PGG and PCC)	O'Kane et al. (2019)	Phase 2 clinical trial	Germline	3/4
SDHC	Tyrosine Kinase Inhibitors	Sensitivity (RCC)	Shuch et al. (2016)	Case Report	Germline	2/4
MLH1	Topoisomerase II inhibitors	Resistance	Fedier et al. (2001)	In vitro cell lines	Tumor derived	2/4
			Aebi et al. (1997)	In vitro cell lines	Tumor derived	
	Topoisomerase I inhibitors	Resistance	Fedier et al. (2001)	In vitro cell lines	Tumor derived	2/4
	Platinum agents	Resistance (Cisplatin and Carboplatin)	Aebi et al. (1997)	In vitro cell lines	Tumor derived	2/4
			Martin et al. (2008)	Review	Not specific	
			Fink et al. (1997a)	In vitro and in vivo	Tumor derived	
			Li et al. (2018)	In vitro and in vivo	Tumor derived	
		Sensitivity (Oxaliplatin)	Fink et al. (1997a)	In vitro and in vivo	Tumor derived	2/4
			Vaisman et al. (1998)	In vitro cell lines	Tumor derived	
	5-Fluorouracil	Resistance	Carethers et al. (1999)	In vitro cell lines	Tumor derived	2/4
			Meyers et al. (2001)	In vitro cell lines	Tumor derived	
			Sargent et al. (2010)	Pooled analysis	Not specific	
	Alkylating agents	Resistance	Taverna et al. (2000)	In vitro, human cell lines	Tumor derived	2/4
	Aspirin and NSAIDs	Sensitivity	Burn et al. (2020)	10-years follow-up of randomized controlled trial	Germline	4/4
			Ouakrim et al. (2015)	Observational study	Germline	
			Reyes-Uribe et al. (2021)	Phase 1 clinical trial	Germline	
MSH2	Topoisomerase II inhibitors	Resistance	Fedier et al. (2001)	In vitro cell lines	Tumor derived	2/4
			Aebi et al. (1997)	In vitro cell lines	Tumor derived	
	Platinum agents	Resistance (Cisplatin and Carboplatin)	Aebi et al. (1997)	In vitro cell lines	Tumor derived	2/4
			Martin et al. (2008)	Review	Not specific	
			Fink et al. (1997a)	In vitro and in vivo	Tumor derived	
			Goodspeed et al. (2019)	CRISPR screen in cell line	Tumor derived	
		Sensitivity (Oxaliplatin)	Fink et al. (1997a)	In vitro and in vivo	Tumor derived	2/4
	5-Fluorouracil	Resistance	Carethers et al. (1999)	In vitro cell lines	Tumor derived	2/4
			Sargent et al. (2010)	Pooled analysis	Tumor derived	
	Alkylating agents	Resistance	Taverna et al. (2000)	In vitro, human cell lines	Tumor derived	2/4
			Friedman et al. (1997)	In vivo models	Tumor derived	
	Aspirin and NSAIDs	Sensitivity	Burn et al. (2020)	10-years follow-up of randomized controlled trial	Germline	4/4
			Ouakrim et al. (2015)	Observational study	Germline	
			Reyes-Uribe et al. (2021)	Phase 1 clinical trial	Germline	
			Mcilhatton et al.	In vivo model	Tumor derived	
MSH6	Platinum agents	Resistance (Cisplatin)	Vaisman et al. (1998)	In vitro cell lines	Tumor derived	2/4
		Sensitivity (Oxaliplatin)	Vaisman et al. (1998)	In vitro cell lines	Tumor derived	
	Temozolomide	Resistance	Nguyen et al. (2014)	In vitro and in vivo	Tumor derived	2/4
	Aspirin and NSAIDs	Sensitivity	Burn et al. (2020)	10-years follow-up of randomized controlled trial	Germline	4/4
			Ouakrim et al. (2015)	Observational study	Germline	
			Reyes-Uribe et al. (2021)	Phase 1 clinical trial	Germline	
PMS2	Platinum agents	Resistance (Cisplatin)	Fink et al. (1997b)	In vitro cell lines	Tumor derived	2/4
	Aspirin and NSAIDs	Sensitivity	Burn et al. (2020)	10-year follow-up of randomized controlled trial	Germline	4/4
			Ouakrim et al. (2015)	Observational study	Germline	
			Reyes-Uribe et al. (2021)	Phase 1 clinical trial	Germline	

Summary of gene-drug relationships, with description of references and evidence scoring.

In this review, each of the 23 heritable cancer genes will be introduced and followed by discussion of the associated relationships, including those that are clinically actionable today and other potential investigative therapies. Our results are summarized into three categories: direct gene-drug relationships, shared risk outcomes, and clinical context decision making. First, we report on the extensive work conducted to understand the molecular mechanisms underlying gene variants in conferring sensitivity or resistance to pharmacologic therapies. Secondly, we identified gene variants and therapies sharing similar adverse clinical outcomes and may possess additive risk. Lastly, we touch on two different clinical contexts that may shape a response to a given gene-drug relationship.

## Methods

### Focused Literature Review

For this comprehensive literature review, multiple sources were utilized to provide a complete compilation of available information. A search for each of the 23 genes included in the study was completed on Clinical Pharmacogenomics Information Consortium (CPIC), The Pharmacogenomics Knowledge Base (PharmGKB), Online Mendelian Inheritance in Man (OMIM®), GeneReviews, UpToDate, and Access Medicine. Secondly a PubMed search was completed for each gene in combination with multiple keywords, including (gene) and (pharmacogenomics) (gene) and (therapy) (gene) and (adverse medication) (gene variant) and (therapy) (clinical syndrome) and (pharmacogenomics). In addition to utilizing the United States Food and Drug Administration Table of Pharmacogenomic Biomarkers in Drug Labeling, an additional review of FDA drug labels was conducted using their available online search tool (https://nctr-crs.fda.gov/fdalabel/ui/search). A PubMed search for similarly related topics was also included to provide appropriate discussion in the context of our study. Each significant finding was classified into one of three categories: direct drug-gene sensitivity or resistance, shared risk outcome, or clinical context consideration. The entirety of the review process was conducted from January 2021 through August 2021.

### Clinical Implementation Scale

To succinctly summarize the evidence identified for each of the relationships listed, a four-tier scoring system, Clinical Implementation Scale (CIS), was created by our team. Each relationship was given a score out of 4, with 4/4 representing the most supported evidence (meta-analyses, multiple clinical trials) and possesses the greatest clinical utility today. Scores of 3/4 represent advisable evidence (few clinical trials, supported *in vitro*/vivo). Scores of 2/4 represent potential or prospective findings that require further validation (*in vitro* and *in vivo* findings). Lastly, evidence receiving a score of 1/4 indicate shared relationships with few to no definitive studies, though still possess a shared risk or considerable outcome. Our CIS scoring system is visualized below:

4/4 Actionable → clinical relationship Meta-analyses, multiple clinical trials
3/4 Advisable → association is present One/few clinical trials, supported cell data
2/4 Prospective → needs confirmation *In vitro*/*in vivo* cell data
1/4 Unknown → shared risk Limited/no studies investigating relationship

## Results

### APC

Operating within the WNT/beta-catenin (canonical WNT) signaling pathway, altered APC protein expression leads to inappropriate “gatekeeping” of cell homeostasis resulting in overactivation of WNT signal transmission and successive accumulation of beta-catenin in the cytoplasm of cells. Upon translocation to the nucleus, beta-catenin exerts its effect as a gene transcription activator leading to cancer progression (Online Mendelian Inheritance in Man OMIM®, 2021a; Krausova and Korinek, 2014). Germline mutations in the *APC* gene cause Familial Adenomatous Polyposis (FAP), an autosomal dominant colon cancer predisposition syndrome affecting as many as 3.2 people per 100,000 individuals. FAP is known clinically for the development of hundreds to thousands of adenomatous polyps within the colon and is inherited with nearly complete penetrance. Colectomy is considered first line therapy in FAP and is an absolute indication in confirmed colon cancer or in presence of distressing symptoms. Currently, there are no FDA-approved chemopreventive therapies for the treatment of FAP (Jasperson et al., 1998).

#### EGFR-Inhibitors

Current FDA-approved medications for the treatment of metastatic colorectal cancer include two monoclonal antibodies (cetuximab, panitumumab) that selectively inhibit EGFR (epidermal growth factor receptor). A member of the Type I receptor tyrosine kinases, EGFR is a transmembrane glycoprotein known to operate in multiple signaling pathways including vascular endothelial growth factor production, cell growth, and induction of apoptosis. Cetuximab and panitumumab competitively inhibit epidermal growth factor binding, preventing the growth and survival of EGFR-expressing tumor cells (FDA, 2004). Clinical usage of the medication has been limited due to drug resistance to wild-type *RAS* subpopulations. Upon review of additional tumor gene subpopulations, *APC* gene status was identified as a key prognostic indicator of EGFR-inhibitor efficacy. *APC* and *TP53* doubly mutated tumors showed the greatest sensitivity to EGFR-inhibitors, while mutated *APC* tumors also possessed a significant sensitivity compared to wild-type *APC* tumors, increasing the potential for wider clinical implementation in metastatic colorectal cancer therapy (Yang et al., 2019).

#### Tankyrase (TNKS)-Inhibitors

Members of the PARP family, tankyrase enzymes one and two play a key role in WNT signaling and beta-catenin transcriptional activation. Recognized as therapeutic targets, inhibition of these enzymes leads to reduced beta-catenin translocation and subsequent cell death in cells harboring *APC* mutations. Recent work by Schatoff et al. (2019) identified variable response rates to TNKS-inhibitors based upon tumor *APC* genotype. ‘Early’ truncating *APC* mutations were found to be highly resistant to TNKS inhibition, while variants within the mutation cluster region (amino acids 1250–1580) revealed sensitivity. These results differ from earlier studies (Tanaka et al., 2017), which proposed all *APC* variant cells were sensitive to TNKS-inhibitors. TNKS-inhibitors may offer patients possessing germline *APC* variants a unique therapeutic option beyond colon resection.

#### Aspirin and NSAIDs

Both NSAIDs and selective COX-2 inhibitors (COXIBs) are known to reduce the incidence and mortality of numerous cancers. Known primarily as critical enzymes in the eicosanoid pathway, Cyclooxygenase-1 (COX-1) and COX-2 complete the two catalytic steps required for the conversion of arachidonic acid into prostaglandin H2. Aspirin covalently and irreversibly binds to COX-1 and COX-2, while other NSAIDs reversibly inhibit these enzymes preventing the downstream signal from inducing inflammation, fever, and prothrombotic events (Kemp Bohan et al., 2020). Also found to contribute a role in cell cycle progression, increased COX-2 expression resulting from upregulation of the Wnt/B-catenin signaling pathway as a key contributing factor in the formation of gastrointestinal polyps. Thus, COX-2 has become a promising target for patients with FAP (McLean et al., 2008; Nuñez et al., 2011). Celecoxib was the first COXIB to be approved as adjuvant therapy for FAP patients by the Food and Drug Administration in 1999, but follow-up investigations revealed an array of toxicities with prolonged time courses, including gastrointestinal bleeding, renal dysfunction, cardiovascular death, myocardial infarction, and stroke (FDA, 2018; Kemp Bohan et al., 2020). Studies involving the use of aspirin as a chemopreventive therapeutic agent have yielded more promising results. In a randomized control trial, Burn et al. (2011) investigated a 600 mg aspirin daily regimen with and without starch in FAP patients. Though the study revealed no reduction in risk of colorectal polyps, there was a statistically significant reduction in polyp diameter in patients treated for at least 1 year. A recent randomized controlled trial in Japan reported FAP patients experienced safe and effective suppression of recurrence for colorectal polyps greater than 5.0 mm when treated with low dose aspirin (Ishikawa et al., 2021). Similarly, a recent meta-analysis estimates a nearly 20% reduction in mortality for all cancer patients who take aspirin (Elmwood et al., 2021). Additionally, for FAP patients experiencing duodenal polyps, a phase II study using a treatment regimen of sulindac and erlotinib resulted in a 71% reduction in duodenal polyp burden, while simultaneously reducing colorectal adenoma burden (Samadder et al., 2018). Aspirin represents a potentially viable option for chemopreventive treatment for FAP patients due to its suppressive effects on large polyp recurrence, mortality reduction, and well tolerated long-term safety profile.

### BRCA1 and BRCA2

Operating within the homologous repair pathway, altered BRCA1/2 protein expression contributes to mismatched recruitment and protein complex formation leading to disrupted repair of double stranded DNA breaks (Cousineau et al., 2005). Mutations in the *BRCA1* or *BRCA2* genes result in the clinical presentation known as hereditary breast and ovarian cancer syndrome. It is estimated *BRCA1* and *BRCA2* mutation prevalence falls between 0.3% and 0.8%, while the prevalence of founder mutations may be even greater in certain populations (Paluch-Shimon et al., 2016). Estimated lifetime risk for developing breast cancer in individuals harboring these germline variants is upwards of 72% and 69% for *BRCA1* and *BRCA2* respectively. Similarly, the lifetime risk of developing ovarian cancer is up to 44% for *BRCA1* and 17% for *BRCA2* variants (Kuchenbaecker et al., 2017). Testing for unaffected *BRCA1* and *BRCA2* carriers has enhanced surveillance and prophylactic procedures (risk-reducing mastectomy and risk-reducing bilateral salpingo-oophorectomy) (Petrucelli et al., 1998).

#### Oral Contraceptives

The protective role of oral contraceptives (OCPs) in the risk reduction of ovarian cancer has been well documented in the general population. Studies investigating the role of *BRCA1/2* in response to oral contraceptives revealed a similar but weaker risk reduction compared to the general population. Despite the protective effects, *BRCA1/2* germline carriers are in a unique clinical conundrum as the use of OCPs possesses a theoretical increased risk of breast cancer. Because studies are unable to fully exclude an increased risk of breast cancer, use of OCPs must be carefully considered within the clinical context (Narod et al., 2002; McGuire et al., 2004; Whittemore et al., 2004; Iodice et al., 2010; Huber et al., 2020).

#### Clomiphene Citrate

Used clinically as an ovulatory stimulant, clomiphene citrate possesses an FDA label warning regarding prolonged usage increasing the risk of borderline or invasive ovarian tumor development. Though no direct study comparing the risk of clomiphene citrate and *BRCA1/2* germline variants exists, epidemiologic studies support these findings in the general population. Because *BRCA1/2* carriers already possess an underlying risk for ovarian cancer, use of clomiphene citrate in these patients must be carefully monitored and considered on a patient-by-patient basis (FDA, 2012; Reigstad et al., 2017).

#### Estrogen-Based Hormone Replacement Therapy

In order to reduce the risk of breast and ovarian cancer, *BRCA1/2* germline carriers are encouraged to undergo risk-reducing bilateral salpingo-oophorectomy (rrBSO) upon completion of childbearing, leading to the induction of surgical menopause. Following surgery patients may experience postmenopausal symptoms, an indication for hormone replacement therapy. Studies have revealed that *BRCA1/2* carriers possess no change or an even further decrease in breast cancer risk while using short-term HRT to control symptoms following surgical menopause (Eisen et al., 2008; Domchek et al., 2011; Kotsopoulos et al., 2016; Gordhandas et al., 2019). In summary, HRT remains contraindicated prior to prophylactic rrBSO in *BRCA1/2* carriers due to an increased breast cancer risk. Following prophylactic rrBSO, short term use of HRT is reasonable and has not been demonstrated to increase risk for breast cancer. Long term use of HRT following prophylactic rrBSO is not advised.

#### Platinum Compounds and TNBC

Great strides have been made in delineating the most effective treatment method for triple negative breast cancer, tumors which are estrogen receptor (ER), progesterone receptor (PR), and HER2 negative. Further investigation has also identified a key relationship for platinum-based chemotherapies in patients harboring *BRCA1/2* germline variants who develop TNBC. Multiple clinical studies reveal improved sensitivity and pathologic complete response to neoadjuvant cisplatin or carboplatin (Silver et al., 2010; Byrski et al., 2014; Hahnen et al., 2017; Caramelo et al., 2019). Similarly, TNBC patients with *BRCA1/2* germline variants experienced significantly improved response to carboplatin when compared to the standard of care docetaxel treatment regimen (Tutt et al., 2018). The use of platinum-based agents provides patients with *BRCA1/2* germline variants with triple negative breast cancer new avenues for future treatment regimens.

#### PARP-Inhibitors (PARPi)

This class of medications is currently FDA approved for advanced, previously treated ovarian cancer with germline BRCA1/2 variants and previously treated metastatic HER2-negative *BRCA1/2* variant breast cancer. Additionally, olaparib possesses approval for first-line maintenance therapy in *BRCA* variant ovarian cancer as well as approval for maintenance therapy of *BRCA1/2* variant pancreatic cancer (Robson et al., 2017; Litton et al., 2018; FDA, 2020a; Madariaga et al., 2020). PARP enzymes are crucial in repair of single-stranded DNA breaks, thus inhibition of these enzymes allows these breaks to persist. *BRCA1/2* carriers are incredibly sensitive to PARP-inhibitors, as single-strand breaks are coupled with the failed double-strand DNA repair leading to cell cycle arrest or cell death. (Farmer et al., 2005). Multiple clinical trials have investigated the efficacy of PARPi as monotherapy and in conjunction with other chemotherapies, with promising disease-free survival and progression free survival data (Madariaga et al., 2020). In a phase III trial, germline *BRCA*-variant pancreatic cancer patients who were treated with maintenance olaparib experienced prolonged progression-free survival compared to placebo (Golan et al., 2019). Promising safety and efficacy profiles have also been shown in rucaparib-based regimens for *BRCA1/2* variant pancreatic cancer (Reiss et al., 2021). As clinical studies continue to delineate the role of PARPi in cancer therapy, this class of medications provides clinicians treating patients with germline *BRCA1/2* variants a vast potential of clinical utility.

### STK11

Functioning as the primary control mechanism for AMP-activated protein kinase (AMPK) family members, STK11 regulates critical cellular mechanisms including metabolism, homeostasis, apoptosis, and cell polarity (The Human Protein Atlas, 2021f). Peutz-Jeghers Syndrome (PJS) is an autosomal dominant disease resulting from heterozygous pathologic variants in *STK11* possessing nearly 100% penetrance. PJS presents clinically with hamartomatous polyps dispersed throughout the gastrointestinal tract in association with pigmented mucocutaneous macules around the mouth, eyes, nostrils, buccal mucosa, and perianal region. Disease prevalence has been difficult to ascertain, occurring in an estimated range of one in every 25,000 to 280,000 individuals (Tchekmedyian et al., 2013). Due to STK11 role as a tumor suppressor, patients with PJS also possess a significant risk for developing an array of malignancies including colorectal, breast, gastric, small bowel, pancreatic, ovarian, cervical, lung, thyroid, and gonadal tumors (McGarrity et al., 2001; Syngal et al., 2015).

#### PD-1 axis Inhibitors

Programmed death ligand 1 (PD-L1) expression is currently the only FDA approved biomarker in advanced lung adenocarcinoma, acting as a prominent therapeutic target. *STK11* variants have been shown to down regulate PD-L1 expression, rendering PD-1 axis inhibitors futile. Skoulidis et al. (2015) first identified *STK11* tumor variants as a key initiator in resistance to PD-1 axis inhibitors while co-occurring with KRAS mutations. In follow-up to their findings, Skoulidis et al. (2018) formalized the specific role of *STK11* in driving resistance in KRAS- mutated lung adenocarcinoma and hypothesized similar findings for *STK11* variants regardless of KRAS status. Recent studies have expanded this hypothesis, as *STK11* variants with wild-type KRAS also confer resistance (Laderian et al., 2020; Schoenfeld et al., 2020). Though further clinical investigation is needed, PD-1 axis inhibitors are likely to be less effective in patients possessing *STK11* variants.

#### ERK Inhibitors

In 2018 Ulixertinib, a ERK1/2 inhibitor, was the first of its class to undergo Phase one study in MAPK mutant advanced solid tumors (Sullivan et al., 2018). This medication is highly potent and selective, resulting in reversible ATP-competitive inhibition of ERK one and 2. Using *STK11* variant NSCLC cell lines and engineered mouse models, Caiola et al. (2020) found tumors with *STK11* mutants expressed distinct sensitivity to ERK inhibitors. Though ERK inhibitors have yet to be studied clinically in patients with deleterious *STK11* variants, these findings are encouraging for further investigation. ERK inhibitors may offer a promising therapeutic option for patients harboring *STK11* variants in the future.

### TSC1 and TSC2

Operating within the mTOR cellular signaling pathway, altered hamartin (TSC1) or tuberin (TSC2) protein expression leads to unregulated activation of mTORC1, a key controller of anabolic cell growth and proliferation (The Human Protein Atlas, 2021g). Tuberous Sclerosis Complex (TSC) is an autosomal dominant disease resulting from heterozygous pathologic variants in either *TSC1* or *TSC2*. Present in approximately one in 5,800 individuals, TSC predisposes patients to a wide array of clinical features involving the skin, brain, kidneys, eyes, heart, and lungs. (Northrup et al., 1999).

#### Estrogen-Based Medications and Contraceptives

Evidence has shown increased levels of estrogen can contribute to lymphangioleiomyomatosis (LAM) induction or progression. Because individuals with pathogenic *TSC1/2* variants have an increased baseline risk for LAM, it is advised that estrogen-containing products be limited or avoided in these patients (Yano, 2002; Oberstein et al., 2003). Though no direct clinical relationship has been defined between *TSC* variants and estrogen products, studies have revealed the promotion of invasiveness and survival of tuberin-null cells when treated with estrogens (Yu et al., 2004; Yu et al., 2009).

### TP53

The “guardian of the genome”, p53 responds to cellular stressors by initiating cell cycle arrest to allow for DNA replication and repair. p53 also contributes to apoptosis, autophagy, senescence, differentiation, antioxidant stress response, and cellular metabolism. In non-stressed cells, p53 activity is repressed through negative feedback binding by ubiquitin ligase MDM2 (Toledo and Wahl, 2006; Bourdon, 2007; Vousden and Lane, 2007). Li-Fraumeni spectrum disease (LFSD) is a high-risk cancer predisposition syndrome with patients developing a range of both childhood and adult-onset malignancies. Inherited in an autosomal dominant pattern, LFSD results from pathologic variants in *TP53* with 7–20% of mutations developing *de novo* (Schneider et al., 1999). Though not well established, one study estimates disease prevalence at one in every 3,555 to 5,476 individuals (de Andrade et al., 2019). Lifetime cancer risk in LFSD is greater than 70% for men and greater than 90% for women. Unlike other cancer syndromes LFSD is not localized to site-specific cancers, manifesting clinically as a heterogeneous collection of cancers with the most common including soft tissue sarcomas, adrenocortical carcinomas, breast cancer, central nervous system tumors, and osteosarcomas (Guha and Malkin, 2017).

#### Genotoxic Chemotherapy agents

Kasper et al. (2018) identified both genotoxic chemotherapy agents (etoposide) and radiotherapy significantly increased the risk of tumor development in a Li-Fraumeni spectrum disease mice model. Their study also recognized that non-genotoxic agents such as docetaxel, a mitotic spindle poison, did not contribute to further tumor formation. In patients possessing a pathologic *TP53* variant, it is advised that surgical and ablative therapies be given priority while avoiding radiotherapy and genotoxic chemotherapies when possible (Frebourg et al., 2020).

#### Chemotherapy and Chronic Lymphocytic Leukemia

Multiple studies have identified *TP53* mutations and 17p deletions as independent drivers of resistance to chemotherapeutic agents in CLL (Döhner et al., 1995; Hallek et al., 2010). Positive effective results were first observed in studies investigating the use of alemtuzumab (monoclonal antibody-CD52) in CLL patients with poor prognostic features including *TP53* disruptions (Lozanski et al., 2004; Pettitt et al., 2012). This medication was approved as first line therapy for CLL before rebranding of the drug as a multiple sclerosis medication by the manufacturer led to the withdrawal of the license in 2012 (Hallek 2019; FDA, 2021). As a result, new and varied treatment regimens have been investigated with promising outcomes. CLL patients with *TP53* dysfunction have seen much improved but not definitive disease control with small molecule agents such as venetoclax (BCL-2 inhibitor) and ibrutinib (Bruton tyrosine kinase inhibitor), especially when used in combination with rituximab and obinutuzumab or idelalisib (Farooqui et al., 2015; Roberts et al., 2016; Stilgenbauer et al., 2016; Hallek 2019). The use of chemotherapy agents as first line therapies in this patient group is not supported.

#### Chemotherapy and Mantle Cell Lymphoma

*TP53* variants are negative prognostic indicators for clinical outcomes in patients with MCL (Eskelund et al., 2017). *TP53* mutated MCL cells escape eradication by high dose cytarabine in combination with rituximab, a standard treatment for general MCL patients. Patients harboring germline *TP53* mutations who develop MCL may be less likely to benefit from a cytarabine-rituximab combination therapy.

#### Carboplatin and Breast Cancer

In a large cohort study of Chinese women with breast cancer, Sheng et al. (2020) identified patients possessing *TP53* germline variants were more likely to respond to carboplatin-based (with or without anthracycline) neoadjuvant therapy compared to anthracycline or taxane-based regimens. Though future clinical studies are needed, these cohort findings represent a reasonable justification for choosing carboplatin-based therapy regimens in *TP53*-variant breast cancer.

#### 5-FU and Colorectal Cancer

5-FU is a synthetic pyrimidine antagonist included as first-line therapy in colorectal cancer regimens. Once transformed intracellularly, 5-FU metabolites disrupt RNA synthesis, initiate single and double-stranded breaks, and inhibit thymidylate synthase contributing to cellular apoptosis. As both germline and sporadic *TP53* variants increase cellular ability to avoid apoptosis initiation, studies have identified *TP53* null cells as resistant to 5-FU (Aghabozorgi et al., 2020). Patients with *TP53* germline variants who develop colon cancer may be less likely to benefit from 5-FU based therapies.

### PTEN

Operating as a negative regulator of the PI3K/AKT pathway, altered PTEN protein expression leads to uninhibited AKT phosphorylation and subsequent inability to initiate apoptosis (Pezzolesi et al., 2007). PTEN hamartoma tumor syndrome (PHTS) is an autosomal dominant disease resulting from heterozygous pathologic variants in *PTEN*. PHTS encompasses five distinct clinical phenotypes, with each phenotypic syndrome including hamartomatous tumor formation and a germline *PTEN* pathologic variant. The most common phenotype seen in *PTEN* variants is Cowden syndrome, consisting of multiple hamartomas found on skin and GI tract, vascular abnormalities, mucocutaneous features including trichilemmomas, papillomatous papules, lipomas, fibromas, and a severe risk for benign and malignant tumors of the thyroid, breast, and endometrium (Blumenthal and Dennis, 2008). As a result of the wide range and insidious onset of clinical manifestations, the estimated disease prevalence of one in every 200,000 is likely underestimated (Nelen et al., 1999; Yehia and Eng, 2001).

#### Tamoxifen/Raloxifene

Though patients with *PTEN* variants possess an increased risk for breast cancer development, there is no direct evidence to support the use of tamoxifen or raloxifene for breast cancer prevention. Additionally, women with *PTEN* germline mutations have up to 28% lifetime risk for endometrial cancer (Tischkowitz et al., 2020), for which these agents are known to increase the risk. Physicians may consider alternative agents in patients with *PTEN* variants in order to limit the potential increased risk of developing endometrial cancer (Yehia and Eng, 2001; Hobert and Eng, 2009).

#### Trastuzumab and Lapatinib

Studies have identified PTEN protein function as a critical component in the therapeutic efficacy of trastuzumab. PTEN is necessary for rapid AKT-dephosphorylation by trastuzumab, contributing to its antiproliferative effect. Evidence also exists for a PTEN-related role in the mechanism of lapatinib, as *PTEN* knockout cell lines showed decreased sensitivity to clinically therapeutic levels of lapatinib (Nagata et al., 2004; Berns et al., 2007; Eichhorn et al., 2008; Wang et al., 2011). Future clinical studies investigating the efficacy of these agents in patients with *PTEN* variants are needed, though initial lab evidence indicates these agents may be less effective.

#### CDK 4/6 Inhibitors

Currently used as part of the standard of care in estrogen receptor-positive HER2-negative advanced breast cancer, CDK4 and CDK6 inhibitors function by preventing complex formation with D-cyclins leading to tumor suppressor retinoblastoma protein (Rb) dephosphorylation and subsequent cell cycle arrest. Both *in vitro* and *in vivo* models of *PTEN* loss revealed increased levels of rephosphorylated Rb and cell cycle progression when treated with CDK4/6 inhibitors. Though the use of CDK4/6 inhibitors in patients with *PTEN* variants needs to be studied further in clinical trials, current evidence does not support successful use of these agents in this patient population (Costa et al., 2020).

#### PI3K-Alpha inhibitors

Typically reserved for advanced breast cancer following CDK4/6 inhibitor resistance, PI3K-alpha inhibitors have been observed clinically and in cell line data to be ineffective when used in *PTEN*-null cells (Juric et al., 2015; Costa et al., 2020). Other agents may be more effective in patients with *PTEN* variants who develop advanced breast cancer.

Each of the described relationships above were identified in studies involving somatic/tumor driven *PTEN* variants and did not involve specific investigation of germline variants. Because breast cancer is one of the most common phenotypes in patients presenting with PHTS, we chose to include the discussions above. The *in vitro* and *in vivo* evidence supports the direct mechanistic link underlying how *PTEN* loss confers resistance to these drugs. Though future clinical evidence is needed relating specifically to germline variant patients, these medications may be less effective in PHTS patients.

### VHL

Operating within the oxygen-sensing pathway, von Hippel-Lindau tumor suppressor protein (pVHL) is recognized as a primary regulator of hypoxia-inducible genes via ubiquitinylation and subsequent degradation of HIF-alpha during times of adequate oxygenation. Disease-causing mutations in *VHL* trigger a hypoxia-analogous condition within cells, leading to excessive levels of HIF-alpha and downstream transcriptional activation of glycolysis and erythropoiesis. Additionally, pVHL also plays a role in a multitude of critical cellular mechanisms including tumorigenesis, cilia formation, post-translational gene expression, apoptosis, and extracellular matrix formation (The Human Protein Atlas, 2021a). von-Hippel Lindau syndrome (VHL) is an autosomal dominant disease resulting from heterozygous pathologic variants of *VHL*, with 20% of these variants developing as *de novo* mutations. Present in approximately one in 36,000 individuals, VHL predisposes patients to an array of both benign and malignant tumors, most notably clear-cell renal cell carcinoma, pancreatic neuroendocrine tumors, and hemangioblastomas in the retina and throughout the central nervous system. Clinical findings and severity are extremely variable, even among families with an identical pathologic variant. Though variable, *VHL* pathologic variants are highly penetrant, as nearly all patients with a confirmed variant are symptomatic by 65 years of age with the mean age of initial presentation of 26 years old (van Leeuwaarde et al., 2018). In addition to known clinical findings, further complications may also occur as angioma and hypertension increase the risk of subarachnoid hemorrhage and cerebellar hemangioblastoma or hypernephroma-like tumors in the kidney predispose patients to polycythemia (Online Mendelian Inheritance in Man OMIM®, 2021b).

#### HIF-2a Inhibitors

Ubiquitously upregulated in *VHL*-variant tumors, HIF-2a is a key proximal signal stimulating tumor growth and has rapidly become a target of interest for potential systemic therapies for VHL patients. Pre-clinical and clinical studies have produced encouraging results, revealing safety and efficacy in patients with advanced clear cell renal cell carcinoma (Chen et al., 2016; Courtney et al., 2020; Choueiri et al., 2021). A phase II study was recently published investigating the objective response of VHL patients with renal cell carcinoma to belzutifan (MK-6482) treatment regimen. Jonasch et al. (2021) reported an objective partial response in 49% of patients, with an additional 49% of patients with stable disease. 96% of all patients experienced progression-free survival at 24 months. Remarkably, the study also reports partial or complete responses in additional VHL-associated neoplasms present within the study population. 77% of patients with pancreatic lesions, 91% of patients with pancreatic neuroendocrine tumors, and 30% of patients with central nervous system hemangioblastomas experienced a response following treatment with belzutifan. Additionally, all retinal hemangioblastomas present in the study population were graded as showing improvement. With only grade I and II adverse events reported, belzutifan has the ability to transform care for VHL patients providing a potential treatment option beyond surgical excision. This systemic therapy targets the underlying pathophysiology of the disease and thus far has shown to effectively halt or reduce VHL-associated tumors throughout the body with minimal side effects.

### MUTYH

The mutant Y homolog *E. coli* (*MUTYH*) gene encodes adenine DNA glycosylase necessary for the single-base excision repair and proper initiation of single-strand DNA breaks following oxidative damage leading to apoptosis (The Human Protein Atlas, 2021b). MUTYH Associated-Polyposis (MAP) is an autosomal recessive disease resulting from biallelic germline pathologic variants in *MUTYH* presenting as homozygotes or compound heterozygotes and occurs in an estimated one in every 20,000 to 60,000 individuals (Al-Tassan et al., 2002; Cleary et al., 2009; Win et al., 2017). Patients with MAP possess a significantly increased risk for developing colorectal polyps and cancer throughout their lifetime. Patients frequently develop between 10 and 100 polyps by the fifth or sixth decade of life, most commonly as adenoma-type, though hyperplastic and serrated polyps have also been identified (Boparai et al., 2008; Grover et al., 2012). It is estimated that 43%–63% of patients develop malignancy by 60 years old, with a median age of onset at 48 years old. MAP has also been reported to increase risk of other malignancies including bladder, ovarian, duodenal, breast, hepatobiliary, gastric, endometrial, and skin cancers (Vogt et al., 2009; Win et al., 2016).

#### Alkylating Agents

Fry et al. (2008) demonstrated that *MUTYH* knockout cells were less responsive upon exposure to a DNA alkylating agent. Due to *MUTYH* role in DNA base repair, alkylating agents are unable to successfully confer cytotoxicity through the formation of DNA crosslinks, escaping cell death. Though typically reserved for use in salvage regimens, alkylating agents play an important role in the treatment of ovarian cancer (Hutchcraft et al., 2021). Further study is necessary to determine whether individuals with ovarian cancer and biallelic germline pathologic variants in *MUTYH* demonstrate this potential resistance to alkylating agents.

### RET

The *RET* gene specifically encodes the RET (Rearranged during Transfection) protein, a transmembrane receptor tyrosine kinase belonging to the cadherin superfamily necessary for cell growth, differentiation, migration, and survival (The Human Protein Atlas, 2021c; Takahashi et al., 1998). Medullary thyroid carcinoma (MTC) is a malignant tumor originating in the parafollicular C-cells of the thyroid resulting from pathologic *RET* variants, marked by drastic increases in calcitonin levels. MTC may be sporadic or hereditary and presents in a range of clinical syndromes known collectively as MEN2. A 2004 study estimates MEN2 occurs in one in every 35,000 individuals (DeLellis et al., 2004). Three clinical subtypes make up MEN2: multiple endocrine neoplasia type 2A and 2B (MEN2A and MEN2B, respectively) and familial MTC (FMTC). Patients with germline *RET* variants are encouraged to avoid dopamine D2 agonists and beta-adrenergic antagonists until pheochromocytoma is sufficiently ruled out, as these agents may trigger adverse events (Eng, 1999).

#### GLP-1 Agonists

Used to improve glycemic control in patients with type II diabetes mellitus, glucagon-like peptide 1 (GLP-1) agonists possess a black-box warning for risk of causing thyroid C-cell tumors. Though the clinical effects in humans are unknown, C-cell tumors developed in dose-dependent and duration-dependent manner in both genders of mice and rats but not primates (Bjerre Knudsen et al., 2010). This class of medications is contraindicated in patients with a personal or family history of MTC or MEN2 syndromes (FDA, 2010). Though studies document a GLP-1 receptor dependent role in C-cell hyperplasia and no association with RET activation, the FDA label remains (Madsen et al., 2012).

#### Tyrosine Kinase Inhibitors

In 2011, the FDA approved the multi-target TKI vandetanib for symptomatic or progressive MTC with unresectable locally advanced or metastatic disease. The phase III study, which showed an objective response in 46.4% of hereditary *RET*-variant MTC, was the beginning of a cascade of investigation into this class of medications (Wells et al., 2012; FDA, 2014). Similarly cabozantinib, also a multi-target TKI, was approved by the FDA for the treatment of progressive, metastatic MTC (Elisei et al., 2013; FDA, 2014). A phase II study involving the use of sunitinib in the treatment of advanced/metastatic paraganglioma and pheochromocytoma found one patient with MEN2A and a germline *RET* variant experienced a 64% tumor volume reduction (O'Kane et al., 2019). Follow up studies involving these and other multi-target TKIs (sunitinib, sorafenib, lenvatinib, anlotinib) have produced a common negative trend, as many of these medications increase progression-free survival without reduction in tumor size and many patients go on to develop disease resistance over time while experiencing a myriad of toxicities (Okafor et al., 2021). As a result, the development and evaluation of highly selective RET-specific TKIs (selpercatinib and pralsetinib) are on the forefront of clinical investigation and are showing promise in preliminary studies. In their phase I/II study, Wirth et al. (2020) revealed a 69% response rate in patients with *RET*-variant MTC who had undergone prior treatment with vandetanib or cabozantinib and a 73% response rate in patients with no prior TKI therapy history when treated with selpercatinib. Subbian et al. (2021) reported similar findings for *RET*-variant MTC patients using a pralsetinib treatment regimen, as 60% of formerly treated patients experienced a response and 71% of patients without prior treatment experienced a response. Both drugs possess a much more tolerable side effect profile than the multi-target TKIs as a result of their specificity. Though isolated reports of acquired tumor resistance drive concerns regarding the long-term efficacy of RET-selective agents, future follow-up studies will be needed to evaluate the viability of prolonged treatment courses. RET-selective TKIs offer patients harboring RET-variant related disease a much needed option for systemic therapy, providing both substantial tumor responses and a tolerable toxicity profile.

### SDHAF2, SDHB, SDHC, SDHD

Succinate dehydrogenase (SDH) is a multi-protein complex functioning in both the tricarboxylic acid cycle and mitochondrial electron transport chain. Four genes required for appropriate assembly and function of SDH include *SDHAF2*, *SDHB*, *SDHC*, and *SDHD*. Hereditary paraganglioma-pheochromocytoma syndrome (HPP) is a tumor predisposition syndrome caused by germline heterozygous pathogenic variants in *SDHAF2*, *SDHB*, *SDHC*, *SDHD*, *MAX*, *SDHA*, and *TMEM127*. HPP is inherited in an autosomal dominant manner in all genes except for *SDHD* and *SDHAF2* which are maternally imprinted (Else et al., 2008). Patients with these genetic variants present clinically with a paraganglioma or pheochromocytoma and are more likely to be multifocal, recurrent, metastatic and occur at an earlier age in comparison to sporadic tumor formation (Lenders et al., 2014). Patients primarily experience symptoms related to catecholamine excess or by mass effect depending on the tumor’s location and ability to secrete catecholamines (Else et al., 2008). Patient phenotypes vary according to which gene is altered. *SDHB* germline variants are most notably associated with the highest morbidity and mortality, commonly developing extra-adrenal sympathetic paragangliomas with the greatest risk for metastatic disease (Andrews et al., 2018).

#### Temozolomide

Used in the management of metastatic paragangliomas and pheochromocytomas, evidence has shown patients with *SDHB* variants respond remarkably well to temozolomide-based regimens (Hadoux et al., 2014). Recent studies in *SDHB*-knockout mouse models showed improved cytotoxicity, reduced metastatic lesions, and prolonged overall survival when treated with temozolomide coupled with a PARP-inhibitor (Pang et al., 2018). The use of temozolomide should be highly considered in patients with *SDHB* variants who develop metastatic paragangliomas and pheochromocytomas.

#### Tyrosine Kinase Inhibitors

TKIs can effectively treat *KIT* or *PDGFRA* mutated gastrointestinal stromal tumors (GISTs). However, *SDHx*-deficient GISTs display a poor response when treated with imatinib and limited response when treated with sunitinib (Boikos et al., 2016). There has been minimal investigation into alternative TKIs (Ibrahim and Chopra, 2020). Though these tumors possess a low overall mortality, *SDHx*-deficient GISTs are prone to progress and recur (Weldon et al., 2017). On the other end of the spectrum, case reports have shown quality responses in SDHx-null renal cell carcinoma to sunitinib and pazopanib (Paik et al., 2014; Shuch et al., 2016). Similarly, the role of TKIs in the treatment of advanced/metastatic paraganglioma and pheochromocytoma is encouraging, with evidence of a patient harboring an *SDHB* variant responding to sunitinib-based therapy (O'Kane et al., 2019). While further studies are needed to support the findings in both disease states, TKIs offer patients with *SDHx*-variant RCC or paraganglioma/pheochromocytoma a potential future therapeutic option when few others exist.

Because of the small sample size most of these studies generalize findings to include all *SDHx* variants. Specifically, Boikos study included *SDHB* and *SDHA*, Shuch included *SDHC*, Paik studied *SHDB*, and O’Kane reviewed *SDHB* and *SDHA*.

### BMPR1A and SMAD4

Bone Morphogenetic Protein Receptor Type 1A (BMPR1A) is a member of the TGF-beta superfamily of receptors, which upon ligand binding forms a receptor complex of transmembrane serine/threonine kinases, triggering downstream SMAD protein activation (Howe et al., 2001). SMAD4 plays a crucial role in transcription regulation and once activated, SMAD family proteins form complexes to bind to and control transcription of target genes (The Human Protein Atlas, 2021d). Juvenile polyposis syndrome (JPS) is an autosomal dominant disease resulting from heterozygous loss of function pathogenic variants in *SMAD4* or *BMPR1A*. JPS presents clinically as a predisposition for developing multiple hamartomatous polyps throughout the gastrointestinal tract. The occurrence of JPS is largely unknown, with an estimated prevalence ranging from one in every 16,000 to 100,000 (Larsen Haidle and Howe, 2003). A variation of this syndrome is present with mixed clinical findings of both JPS and hereditary hemorrhagic telangiectasia including epistaxis, telangiectasia, arteriovenous malformations, and digital clubbing. Patients with *SMAD4* pathogenic variants have also been found to develop thoracic aortic aneurysms and aortic dissections (Wain et al., 2014). On the other hand, gain of function mutations in *SMAD4* have been associated with a different clinical presentation, a complex connective tissue disorder known as Myhre syndrome (Caputo et al., 2012). Symptoms of Myhre syndrome frequently include extensive proliferative fibrosis and intellectual disability, and with phenotypes displaying varying involvement of the cardiovascular, respiratory, gastrointestinal, and skin systems (Starr et al., 2021).

#### 5-Fluorouracil

First recognized as a poor prognostic factor in colorectal cancer, more recent studies have identified a correlation between SMAD4 expression and 5-FU sensitivity. In patients with colorectal cancer treated with 5-FU, those with *SMAD4* loss displayed a significant decrease in both overall survival and progression free survival (Alhopuro et al., 2005; Papageorgis et al., 2011; Wasserman et al., 2019). For patients harboring *SMAD4* variants and requiring adjuvant chemotherapy, options other than 5-FU may likely be more effective.

#### Cetuximab

Multiple studies have established an association between SMAD4 protein expression and sensitivity to cetuximab therapy. Lin et al. (2019) showed *SMAD4* knockdown tumor cell lines were more viable and inhibited cell apoptosis when treated with cetuximab in comparison to cells with overexpression of SMAD4. Similar findings were observed in somatic *SMAD4* loss of head and neck squamous cell carcinoma, conferring resistance to cetuximab therapy (Ozawa et al., 2017). Other agents are likely to be more successful than cetuximab in treating germline SMAD4 variant pathologies.

#### Irinotecan

Wong et al. (2020), while also conferring resistance of *SMAD4*-negative cell lines to 5-FU, found these same lines also conferred resistance to irinotecan. Conversely, the study found no resistance to therapeutic oxaliplatin levels. Patients with colorectal cancer harboring *SMAD4* pathogenic variants may obtain more benefit from adjuvant chemotherapies that do not include irinotecan.

#### Fluoroquinolones

Patients harboring *SMAD4* variants are known to possess an increased risk for collagen-associated disorders, including aortic aneurysm or aortic dissection (Meng et al., 2019; Yu et al., 2019). Though no direct relationship has been established between fluoroquinolones and *SMAD4* pathogenic variants, both are associated with an increased risk for aortic aneurysms and dissections. Because patients with pathogenic *SMAD4* variants commonly have one or more features of hereditary hemorrhagic telangiectasia (HHT), these patients may potentially increase their risk for developing severe aortic symptoms when taking fluoroquinolones.

### MLH1, MSH2, MSH6, PMS2

Key in initiating DNA repair, MSH2 and MSH6 form the heterodimer MutS alpha which recognizes and binds to mismatched dsDNA. Following binding, MutS alpha recruits a second heterodimer MutL alpha formed by MLH1 and PMS2. The coupled complex activates the endonuclease activity of PMS2 to induce single strand breaks and recruits DNA polymerase III to the site of DNA mismatch (The Human Protein Atlas, 2021e). Lynch Syndrome is an autosomal dominant disease caused by heterozygous germline mutations in *MLH1*, *MSH2*, *MSH6*, *PMS2* or an *EPCAM* deletion, occurring in one of every 279 individuals (Win et al., 2017). Characterized primarily by an increased risk for colorectal cancer, Lynch Syndrome also increases the risk of cancers of the endometrium, ovaries, stomach, small bowel, urinary tract, brain, pancreas, and prostate (Dominguez-Valentin et al., 2020). Patients present with unique clinical phenotypes correlating to each of the associated Lynch Syndrome genes. *MLH1* variants possess the greatest risk for colorectal cancer, with evidence supporting a younger age of onset and increased risk for developing multiple primary tumors (Pinto et al., 2018). Conversely, *MSH2* variants are frequently associated with extracolonic cancers and are more likely to present as the Muir-Torre version of Lynch Syndrome (Everett et al., 2014).

#### Topoisomerase Inhibitors

Fedier et al. (2001) found *MLH1* and *MSH2*- deficient cell lines conferred resistance to topoisomerase II inhibitors (epirubicin, doxorubicin, and mitoxantrone). *MLH1* loss has also been demonstrated to display resistance to topoisomerase I poisons. Etoposide resistance was also identified in previous studies in using *MLH1* and *MSH2* deficient colorectal and endometrial carcinoma cell lines respectively (Aebi et al., 1997). For patients possessing germline *MLH1* or *MSH2*, use of topoisomerase-blocking agents are less likely to produce cell death.

#### Platinum Agents

Binding to DNA and creating DNA adducts, platinum agents produce crosslinks disrupting the DNA helix. These agents rely on a proper functioning mismatch repair system to identify damaged DNA and induce apoptosis. Studies have documented the role of *MLH1* and *MSH2* loss in the development of cisplatin and carboplatin resistance in colorectal carcinoma, as well as *PMS2* loss conferring low level cisplatin resistance (Aebi et al., 1997; Fink et al., 1997a; Fink et al., 1997b; Martin et al., 2008). Similar resistance to cisplatin was also found in *MLH1*-silenced endometrial cell lines and *MSH2*-silenced bladder cancer cell lines (Li et al., 2018; Goodspeed et al., 2019). On the other hand, evidence has shown colorectal cancer cells possessing disrupted *MLH1* and *MSH6* confer similar sensitivity to oxaliplatin as wild-type cells (Fink et al., 1997a; Vaisman et al., 1998). When initiating chemotherapy regimens that include platinum agents, patients harboring *MLH1* and *MSH2* variants may be more successful with oxaliplatin over cisplatin or carboplatin-based regimens.

#### 5-Fluorouracil

Though not all studies agree, most clinical findings indicate *MLH1* and *MSH2* deficiencies contribute to poorer outcomes with 5-FU treatment for colorectal cancer (Ribic et al., 2003; Sargent et al., 2010). Similar findings have been reported in multiple cell line studies, supporting evidence of resistance to 5-FU (Carethers et al., 1999; Meyers et al., 2001). Typically used as the standard of care for stage II and III colorectal cancer, 5-FU based regimen may not be as effective in patients with *MLH1* and *MSH2* variants.

#### Alkylating agents

Multiple studies have evaluated and identified high degrees of resistance from *MLH1*, *MSH2*, and *MSH6* deficient tumor cell lines to temozolomide (Friedman et al., 1997; Taverna et al., 2000; Nguyen et al., 2014). Evidence for other alkylating agents such as cyclophosphamide and busulfan have also been implicated, though results are not consistent. Though further studies are needed to specifically address the role of these agents in patients with germline variants, the aforementioned studies present evidence of resistance in patient tumor samples indicating that these agents may be less likely to be effective.

#### Aspirin and NSAIDs

Like FAP patients, Lynch Syndrome patients have also seen quality outcomes when treated with aspirin and NSAIDs. In a long-term follow-up to their 2011 CAPP2 study, Burn et al. (2020) showed that aspirin provides significant protective effects against colorectal cancer in Lynch Syndrome patients for an upwards of 20 years. Daily aspirin use for at least 2 years produced a reduced hazard ratio of 0.65 and 0.56 for intention-to-treat and per-protocol analysis, respectively. Ouakrim et al. (2015) produced related results when investigating both aspirin and ibuprofen. In their study, Lynch Syndrome patients treated with aspirin for 1 month were estimated to experience a 60% decrease in colon cancer risk and a 65% decrease in risk when treated with 1 month of ibuprofen. A recent phase I study found naproxen was safe for chronic use as a primary and secondary chemopreventative intervention at both low and high doses in Lynch syndrome patients. Additionally, a co-occurring tissue-specific mouse model showed naproxen modulated tumor growth and prolonged survival (Reyes-Uribe et al., 2021). Currently underway are two additional randomized clinical trials, CAPP3 and AAS-Lynch, which share a primary endpoint of comparing the effectiveness of varying aspirin doses in preventing colorectal cancer in Lynch Syndrome patients (Soualy et al., 2020).

## Discussion

This investigation into the relationships existing between genetic data and therapeutic outcomes is the tip of the iceberg in the continued pursuit of personalized medical decision making. The genes presented above represent many of the most common cancer predisposition syndromes, each with potentially actionable pharmacologic considerations. In total, 54 relationships were identified and scored according to our confidence scale (Table 1). Our work goes hand-in-hand with two National Institute of Health initiatives already underway: CPIC and PharmGKB. Both initiatives provide peer-reviewed, evidence-based translation of research findings into clinically actionable decision making in gene-drug pairs. Our review works to build on the understanding of gene-drug relationships by also considering the disease component, as clinical phenotypes also shape potential interactions.

Currently, CPIC has published clinical decision-making guidelines for more than 440 unique gene-drug relationships, largely consisting of drug metabolizers and transport genes. Of note, CPIC has also developed guidelines for germline variants of *RYR1* and *CACNA1S*, presenting clinically as malignant hyperthermia (inherited, familiar syndrome). The publication of these genes marks a giant leap forward in developing gene variant-specific clinical decision support. Our study hopes to contribute to this area of expertise, as CPIC does not yet have any existing guidelines created for the disease genes investigated in our study. There is also great potential for investigation into other non-cancerous germline familial syndromes, specifically relating to cardiology and inborn errors of metabolism.

Similarly, of the 100 different pharmacologic agents listed in the United States Food and Drug Administration Table of Pharmacogenomic Biomarkers in Drug Labeling, only eight drugs include labeling for any of the 23 genes included in this study (also described in Table 2). By taking a broad and pre-emptive approach, our study was able to include relationships at the gene level, make connections to other FDA drug label associations, and provide discussion on difficult clinical context decision making.

**TABLE 2:** PharmGKB drug label annotations.

Gene	Drug class	Relationship	Drug	FDA/EMA label	Evidence score/Summary
BRCA1/2	PARP-inhibitors	Sensitivity	Niraparib	FDA: ZEJULA	Testing required
			Olaparib	EMA: LYNPARZA	Testing required
				FDA: LYNPARZA	
			Rucaparib	EMA: RUBRACA	Testing required
				FDA: RUBRACA	
			Talazoparib	EMA: TALZENNA	Testing required
				FDA: TALZENNA	
RET	Tyrosine Kinase Inhibitors	Sensitivity	Vandetanib	EMA: CAPRELSA	Testing recommended
			Pralsetinib	FDA: GAVRETO	Testing required
			Selpercatinib	FDA: RETEVMO	Testing required
			Cabozantinib	FDA: COMETRIQ	Informative PGx
TP53	BCL-2 Inhibitors	Sensitivity	Venetoclax	FDA: VENCLEXTA	Informative PGx

Description of current drug label annotations for genes investigated in this study, including links to drug label. Results were obtained from PharmGKB.

As the field of pharmacogenetics continues to progress, clinical use of genomic information has made precision medicine an everyday reality. Yet, much of the available clinically actionable pharmacologic cancer specific evidence pertains to somatic or secondary treatment resistant tumor markers. In 2014, Wheeler et al. was a leader in reporting specifically on cancer pharmacogenomics, delineating evidence for gene-drug relationships including genes *TMPT*, *UGT1A1*, *CYP2D6*, and *SLC O 1B1*. Pasternak et al. (2017) investigated how germline variants affect disease outcome and severity as well as drug response. Their work summarized available evidence to provide discussion on eight individual pharmacogenes and their related drug implications. Lauschke et al. (2017) also provided a similar overview of genomic biomarkers as predictors of drug response and included approved targeted cancer drugs. Wellmann et al. (2018) investigated similar ends through a different approach, reviewing 125 oncology drugs for positive germline pharmacogenomic associations. Their work resulted in 12 individual clinical decision support summaries, six for prior established FDA pharmacogenomic associations and six for novel relationships. Our study set out to build upon these studies from a unique and alternative perspective, comprehensively review existing literature on an individual gene-by-gene basis. Our thorough investigation specifically focused on germline variants possessing a cancer-predisposition and included discussion of both current and prospective relationships.

Our study also included a comprehensive review of the *MEN1* and *RB1* genes, which yielded limited evidence of pharmacologic relationships with these germline variants. As mentioned below, evidence shows each of these genes possess an increased sensitivity to radiation which may help guide clinical decision-making. Further investigation into potential therapies, clinical outcomes, and pharmacologic sensitivities for patients with these conditions is warranted.

One complex relationship shared among many of the cancer predisposition genes reviewed in this study involves the role of radiation-based imaging and radiation-based therapy. Studies revealing poor outcomes following radiation exposure exist for patients with germline mutations in *TSC1/2*, *TP53*, *MUTYH*, *SMAD4*, *MLH1*, and *RB1*, with additional recommendations for conservative approaches taken in *MEN1*, *STK11*, and all pediatric populations (Wong et al., 1997; Matsumura et al., 1998; Evans et al., 2006; Tokairin et al., 2006; Kleinerman et al., 2007; Kleinerman 2009; Lenders et al., 2014; Wang et al., 2018; Lavergne et al., 2020). Limiting radiation exposure is a well understood clinical practice. Our findings offer evidence that patient populations with specific underlying germline variants are more sensitive to radiation exposure than the general public, requiring careful clinical consideration and observation for secondary primary tumor development following exposure. Many recommendations for surveillance scans with MRI or nuclear-based imaging over radiation-based exist. The use of radiation therapy is not uniform, as clinical usage varies upon patient presentation, tumor pathology, and tumor location. In many circumstances, surgical management must first be ruled out prior to the use of radiation therapy in cancer predisposition patients.

An additional but separate finding related to our study involves the delicate relationship between multiple pharmacologic agents and pheochromocytoma. As a catecholamine producing tumor, pheochromocytomas have the ability to produce severe adverse hemodynamic effects in the presence of surgical anesthesia, beta-receptor antagonists, dopamine receptor antagonists, sympathomimetics, antidepressants, and corticosteroid hormones (Eisenhofer et al., 2007). FDA labels for metoclopramide and prochlorperazine each include contraindications for use in the setting of a pheochromocytoma, as case clinical reports the development of hypertensive crisis in these patients. Though this relationship is not specifically related to a germline variant, *RET*, *SDHB*, *SDHC*, *SDHD*, and *SDHAF2* each include a predisposition of developing pheochromocytoma and thus must be considered. Evaluation and ruling out of pheochromocytoma prior to initiating therapy with any of the agent discussed can prevent serious adverse events in these patients.

The greatest strength of our study was the uniqueness of our approach. We believe our study was the first of its kind to specifically seek out both prospective and current evidence for gene-drug relationships on a gene-by-gene basis. This approach allowed us to thoroughly investigate each of the 23 oncologic genes listed in the ACMG59, to ultimately provide clinicians with a succinct and comprehensive discussion on the potential gene-drug relationships for their patients. There is also great potential to build upon our study, as similar gene-drug relationships exist in cancer predisposition genes beyond the scope of the ACMG59 list. The NCCN guidelines include additional genes, such as *PALB2*, which has been investigated alongside *BRCA1/2*. Clinical trials recognize germline *PALB2* variants as a sensitive target for PARP-inhibitors in platinum-sensitive advanced pancreatic cancer (Reiss et al., 2021).

We also recognize our study possesses some limitations. Due to the rarity of many of the investigated germline variants, few large-scale clinical trials exist. A large majority of the included studies involve cohort investigations of regionally based populations. Additionally, as mentioned above, some clinical relationships are expanded from evidence involving tumor-collected variant studies. These studies were included on the basis that the germline variant syndrome possesses an increased risk of developing a similar clinical phenotype as the study, and thus by harboring a similar genetic variant is likely to experience a related outcome.

## Conclusion and Future Approaches

The future of genetic information in the clinical setting is an exciting and challenging opportunity. Through further investigation into the wide expanse of genetics, we can continue to drive forward the ability to generate highly individualized DNA-level care for all patients. By continuing to work collaboratively with evidence-based organizations, we can help provide clinicians with the critical information they need to best treat their patients. Future studies will be needed to validate and further delineate therapies and outcomes specific to genetic variants. Clinical trials differentiating outcomes based upon genetic variants within the study population will be needed to build upon existing evidence. Similarly, as further data is uncovered, clinical decision support documentation will be needed to provide clear and definitive language for providers.
